# Pamphlets Received

**Published:** 1855-04

**Authors:** 


### EDITORIAL DEPARTMENT.

---

### *Pamphlets Received*

*On Injection of the Bronchial Tubes, and Tubercular Cavities of the Lungs.* By HORACE GREEN, M. D., LL. D.

It is well known to our readers that Dr. Green has taken the lead in the local treatment of diseases of the throat and air-passages, and if not the first, was certainly one of the first to carry the sponge-probang through the rima-glottidis and into the trachea. It was for a long time a matter of dispute whether this was anatomically possible, but we believe that the point is now generally conceded, and certainly very many honest men are greatly deceived in their own actions, if this operation is not daily achieved in all parts of our country. This belief has not obtained, however, without a vigorous contest, in which personal feeling has had an unfortunate share. Dr. Green has been, and to a great extent still is, "the best abused man" in the profession.

We have never ranked among the opposers of Dr. Green's views. On the contrary, we have uniformly asserted the practicability of his means of treatment. In the objections we may offer to this present pamphlet, we must not be ranked among those who oppose the author, rather than his theory. If Dr. Green can satisfy some doubts we now have as to his late achievements in catheterization of the air-passages, we shall rejoice in his success.

On page eight of the pamphlet we find the following:

"Another patient was selected for repeating the experiment. This gentleman was the Rev. Mr. McAnn, the superior third of whose epiglottis could be seen easily by depressing the tongue. Upon the laryngeal face of this cartilage, the extremity of the tube was placed and introduced readily through the chink of the glottis into the trachea. This operation was also performed in the presence of several physicians, among whom were Dr. Sims, Prof. E. H. Parker, and several others. The instrument being thus introduced, one of the physicians closed the anterior nares of the patient, and the light was again extinguished by the expired air through the tube. A large pasteboard card, perforated in the center, and of sufficient size to screen the nose and mouth completely, was then slipped over the tube, to which it fitted closely, and the patient directed to blow out the light, which was accomplished through the tube as promptly as in the first instance. After all this, Mr. McAnn was requested to expand the chest, by breathing through the instrument. This was several times performed—the patient inhaling and exhaling easily and freely through the inserted tube. These experiments were subsequently repeated on some eight or ten patients, always with the same results, and in each instance in the presence of several medical men."

This is possible, and we have no right to doubt it when we consider the character of the witnesses. Besides there are no serious anatomical objections. If this were all, we should concede the whole matter, but on the succeeding page Dr. Green proves too much. After mentioning a large number of most respectable medical names as witnesses of this first operation, he gives the following experiment, to which no references are attached:

"In order to test, still further, the truth of the operation, a small air-tight elastic bag was tied over the upper extremity of the tube, and on introducing the instrument, six or eight inches into the trachea of a gentleman, this little bag was inflated and collapsed a dozen times, by the acts of inspiration and expiration on the part of the patient. In performing this last experiment, an incident occurred (which, had the tube been shorter, might have proved an accident,) that is an additional proof of the position of the instrument. The tube which, as I have stated, is thirteen inches long, was introduced its whole length, so that the upper extremity was flush with the lips of the patient, the elastic bag, which is three inches long, only remaining out of his mouth. After the patient had filled and emptied the sac several times, I let go, for a moment, my thumb and finger hold of the extremity of the tube. Just then the patient made a strong inspiration, when the whole instrument, sac and all, was drawn suddenly in, and for a moment disappeared out of sight. Thrusting my fingers immediately into the throat of the patient, I could barely reach, at the base of the tongue, the upper extremity of the bag, which I seized with my thumb and finger, and drew the whole out together."

We are here left to the conclusion (as the bag, "which is three inches long," was drawn in to such a distance that he "could barely reach, at the base of the tongue, the upper extremity of the bag,") that thirteen inches of tube, and two, (at least) of bag, were introduced into the air-passages. But suppose that none of the bag entered the larynx, we must suppose the tracheal and bronchial tubes to be thirteen inches long, and for that distance of a diameter large enough to freely admit the tube.

We will assume the following measurements which we take mostly from Sharpey and Quain:

| | |
|---|---|
| Length of Larynx, - - - - | 2 inches. |
| " " Trachea, - - - - - | $4\frac{1}{2}$ " |
| " " Left Bronchus, - - - | 2 " |
| Total, - - - - - | $8\frac{1}{2}$ " |
| Add for elongation of Trachea, - - | 1 " |
| | $9\frac{1}{2}$ " |

This leaves three and a-half inches of tube to enter the ramifications of the bronchus. As these rami break up very rapidly, and at difficult angles —very frequently right angles—it seems to us that even with the very liberal allowances and measurements we have given, the thing is anatomically impossible. A few experiments on the dead subject would settle the question. So far as we know, none such have been made, except a single one performed by one of our colleagues on the lung of a tubercular subject, with reference to the practicability of injecting a cavity. The lung being removed from the body, a tubercular cavity was laid open, and the pipe of a syringe inserted in the bronchus leading to it. It was found that none of the water contained in the syringe could be forced through into the cavity. Whether repeated experiments would confirm this result is still a question.

We shall await with interest the report of the committee of the New York Academy of Medicine appointed to investigate this point of therapeutics. In the meantime it should not be forgotten that, in event of sufficient proof of the practicability of this operation, we have still left for discussion its availability, its safety, and the more important question of its curative value. That we can do a thing is no reason why we should do it; and it yet remains to be proved that local treatment of tubercular cavities can result in the removal of the phthisical dyscrasia, or can even assist in remedying the destructive action of tubercle upon the lung tissues.

*Anniversary Discourse before the New York Academy of Medicine. Delivered in Clinton Hall, November* 22*d*, 1854. By JOHN H. GRISCOM, M. D. Published by order of the Academy.

We should be glad to republish this very able address. It takes the same view of the position of the Profession which is now so generally attracting the attention of the medical politician.

As a specimen of Dr. Griscom's argument, he states the amount received n board and salary by the physicians—169 in number—attached to the various charitable institutions in New York, at $27,112. Estimating their services, together with the services of physicians to the private poor, at the 'lowest market value," he makes out the following bill:

"January 1st, 1854.

The People of the City of New York,

To the Medical Profession, Dr.

| | |
|---|---|
| To Professional Services rendered in Public Institutions, and to the Poor in private, during the year 1853, - | $835,458 00 |
| Cr. by cash and entertainment, - - - - - | 27,112 00 |
| Balance, - - - - - - | $808,346 00 |

To which we may add,

Payment not expected."

We approve most heartily of the objects of this discourse, and hope that continued discussion may result in some reform of this immense abuse.

*Oration delivered before the Physico-Medical Society of New Orleans, at their Anniversary Meeting, held December*, 1854. By A. MERCIER, M. D., P.

We have read this little brochure with much interest. It is a neat and elegant composition, devoted to the recital of the claims of American physicians and surgeons to distinction, as compared with the medical celebrities of Europe. Dr. Mercier makes an exhibit of this subject in a light which is very flattering to our love of country.

*The Transactions of the New Hampshire Medical Society, (Sixty-fourth Anniversary,) held at Concord, June 6th and 7th*, 1854.

This is a very neat pamphlet, made up of interesting material. The Annual Address by the President, Prof. Albert Smith, was on the subject of "Conservatism in Medicine;" which he handled in a healthy and judicious spirit.

Dr. Andrew McFarland, of the Illinois Insane Hospital, gives an Oration on the "Poetry of the Medical Profession," written in a very fascinating style; and Dr. Wm. H. H. Mason read an able dissertation on the chemical changes that take place in the human body while in a state of disease.

We are indebted to Dr. Hubbard, of the N. H. Journal of Medicine, for our copy of the Transactions. H.

